# Honokiol induces reactive oxygen species-mediated apoptosis in *Candida albicans* through mitochondrial dysfunction

**DOI:** 10.1371/journal.pone.0172228

**Published:** 2017-02-13

**Authors:** Lingmei Sun, Kai Liao, Chengcheng Hang, Dayong Wang

**Affiliations:** 1 Department of Pharmacology, Medical School of Southeast University, Nanjing, China; 2 Department of Pathology and Pathophysiology, Medical School of Southeast University, Nanjing, China; 3 Key Laboratory of Developmental Genes and Human Disease in Ministry of Education, Medical School of Southeast University, Nanjing, China; Universidad Pablo de Olavide, SPAIN

## Abstract

**Objective:**

To investigate the effects of honokiol on induction of reactive oxygen species (ROS), antioxidant defense systems, mitochondrial dysfunction, and apoptosis in *Candida albicans*.

**Methods:**

To measure ROS accumulation, 2′,7′-dichlorofluorescein diacetate fluorescence was used. Lipid peroxidation was assessed using both fluorescence staining and a thiobarbituric acid reactive substances (TBARS) assay. Protein oxidation was determined using dinitrophenylhydrazine derivatization. Antioxidant enzymatic activities were measured using commercially available detection kits. Superoxide dismutase (SOD) genes expression was measured using real time RT-PCR. To assess its antifungal abilities and effectiveness on ROS accumulation, honokiol and the SOD inhibitor N,N′-diethyldithiocarbamate (DDC) were used simultaneously. Mitochondrial dysfunction was assessed by measuring the mitochondrial membrane potential (mtΔψ). Honokiol-induced apoptosis was assessed using an Annexin V-FITC apoptosis detection kit.

**Results:**

ROS, lipid peroxidation, and protein oxidation occurred in a dose-dependent manner in *C*. *albicans* after honokiol treatment. Honokiol caused an increase in antioxidant enzymatic activity. In addition, honokiol treatment induced SOD genes expression in *C*. *albicans* cells. Moreover, addition of DDC resulted in increased endogenous ROS levels and potentiated the antifungal activity of honokiol. Mitochondrial dysfunction was confirmed by measured changes to mtΔψ. The level of apoptosis increased in a dose-dependent manner after honokiol treatment.

**Conclusions:**

Collectively, these results indicate that honokiol acts as a pro-oxidant in *C*. *albicans*. Furthermore, the SOD inhibitor DDC can be used to potentiate the activity of honokiol against *C*. *albicans*.

## Introduction

Most fungal infections in immunocompromised individuals are induced by *Candida albicans*, an opportunistic fungal pathogens [1,2]. Treating *C*. *albicans* infections can be difficult, as there are only a few available classes of antifungals, including echinocandins, polyenes, and azoles [3]. This coupled with increased incidence of candidiasis as well as the rise in drug resistance in *C*. *albicans*, makes the development of novel and effective antifungal agents critically important [3,4]. A common fungicidal mechanism employed by most antifungal drugs involves the production of reactive oxygen species (ROS), such as superoxide anion radicals and hydrogen peroxide [5]. To this end, mitochondria play important roles in energy production, the synthesis of key metabolites, apoptosis regulation, calcium buffering, and in the generation of endogenous reactive oxygen species [6]. Enzymatically, the cellular antioxidant system is comprised of major antioxidant enzymes, including superoxide dismutases (SOD), catalase (CAT), glutathione peroxidase (GPX), and other non-enzymic antioxidants (Vitamin E, vitamin C, carotenoids) [6]. Of these, SOD contributes to virulence in many pathogenic bacteria and fungi [7]. For example, *C*. *albicans* has six SOD enzymes. SOD1-3 are cytoplasmically located and SOD4-6 are glycosylphosphatidylinositol (GPI)-anchored cell wall-associated enzyme [8]. SOD1, SOD4, SOD5, and SOD6 are Cu, Zn-containing SOD which can be inhibited by the Cu, Zn-SOD inhibitor N,N′-diethyldithiocarbamate (DDC) [9].

Small molecules isolated from natural plants can exhibit noticeable antifungal activities and are a promising source for the development of novel antifungal agents [10]. Natural phenolic compounds can be used as potent redox cyclers, which may inhibit microbial growth by disturbing normal redox homeostasis [10]. Previous research has confirmed a clear synergistic effect between certain clinical antifungal agents and phenolic compounds against *C*. *albicans*. This effect is likely due to its ability to induce apoptosis by the increasing ROS levels [11].

Honokiol, a phenolic compound, has been isolated from the bark of the *Magnolia officinalis* tree. It has been used in traditional Chinese medicine due to its potent properties, which include anti-hyperglycemic [12], anti-depressant [13], anti-tumor [14], and anti-inflammatory abilities [15]. Previous work from our lab has shown that honokiol can inhibit adhesion, transition from yeast to hypha, and biofilm formation in *C*. *albicans* [16].

Given this, the present study sought to extend these findings and illustrate the effects of honokiol on inducing reactive oxygen species (ROS), antioxidant defense systems, mitochondrial dysfunction, and apoptosis in *C*. *albicans*. We also investigated the effect of DDC on the antifungal activity of honokiol and production of ROS in *C*. *albicans*.

## Materials and methods

### Materials

Honokiol (5,5’-diallyl-2,4’-dihydroxybiphenyl) was purchased from Xi'an Yuquan Biological Technology Co., Ltd and its purity is over 98% analyzed by high-performance liquid chromatography. DCFH-DA (2′,7′-dichlorofluorescein diacetate), DPPP (diphenyl-1-pyrenylphosphine),JC-1(5,5′,6,6′-tetrachloro-1,1′,3,3′-tetraethylbenzimidazolocarbocyanine iodide), DDC, and other molecular grade agents were obtained from Sigma Chemicals (St. Louis, MO, U.S.A.). Annexin V-FITC apoptosis detection kit, GPX assay kit, CAT assay kit, SOD assay kit, and BCA protein assay kit were purchased from Beyotime Biotechnology (Shanghai, China).

### Microorganisms

*C*. *albicans* wild type strain SC5314 and YEM30, clinical isolates of *C*. *krusei* (CK3), *C*. *tropicalis* (CT2), *C*. *glabrata* (CG1), and *C*. *parapsilosis* (CP1) were used in this study [17]. These clinical isolates were kindly provided by the Shandong Provincial Qianfoshan Hospital, Jinan, China. *C*. *albicans* CAI4-GFP-TOM70 was a gift from Hongxiang Lou (Shangdong University, China) [17]. The yeast strains were cultured in YPD broth (yeast extract 1%, peptone 2%, dextrose 2%). For solid agar plates, 2% bacto agar (Difco, BD Biosciences) was added to the medium. All strains were stored as frozen stock with 15% glycerol at –78°C. Before each experiment, the strains were recovered on YPD plates.

### Antifungal susceptibility testing

The antifungal activities of all tested compounds were performed as previously described [16,18].

### Measurement of ROS production

Endogenous levels of ROS were assayed by a flow cytometer with DCFH-DA staining. Briefly, the cells were adjusted to 1×10^7^ cells/mL in YPD medium and exposed to different concentration of honokiol at 37°C for 4h. The positive control was treated with 1.5mmol/L of hydrogen peroxide at the same condition. After staining with 10 μmol/L DCFH-DA at 37°C for 30 min, the cells were collected and washed three times with PBS. The fluorescence intensities (excitation and emission of 488 and 540 nm, respectively) of cells were tested with a flow cytometer (Becton-Dickinson Immunocytometry Systems, San Jose, CA) and the fluorescence images were taken using a fluorescence microscope with FITC filter (Olympus IX71, Olympus Co., Tokyo, Japan).

### Preparation of cell-free extracts (CFE)

*C*. *albicans* SC5314 cultures were adjusted to 1×10^7^ cells/mL in YPD medium and exposed to different concentration of honokiol at 37°C for 4h. Cell free extract (CFE) of treated and control *C*. *albicans* SC5314 was prepared as described by Khan [19]. After centrifugation, cells were suspended in homogenizing buffer (1 mmol/L phenylmethylsulphonyle fluoride, 250 mmol/L sucrose, 10 mmol/L Tris–HCl, pH 7.5). Glass beads (0.45–0.5 mm diameter) were added to this suspension. CFE were prepared using a FastPrep homogenizer (Fastprep FP120; Savant Instruments, NewYork, USA). The homogenate was collected and centrifuged at 1000g for 5 min at 4°C to remove unbroken cells and glass beads. The pellet was resuspended and recentrifuged with the same homogenizing buffer. The combined supernatants were then centrifuged at 10000×g for 45 min in suspension buffer (10 mmol/L Tris–HCl, pH 7.5) to separate CFE in the supernatant from crude membrane in the pellets. The supernatant thus obtained was used as CFE, whereas the pellet represented the crude membranes. The fraction was aspirated and checked for enzyme activity. Protein quantity was estimated by BCA protein assay kit.

### Cell staining with DPPP

*C*. *albicans* cells were incubated in YPD with or without compounds at 37°C at a density of 1×10^7^/mL for 4h. The cell suspensions were then incubated with 10 μmol/L of DPPP at 37°C for 10 min. The images of fluorescence were taken using a fluorescence microscope with 340/380 nm excitation filter. The fluorescence positive cells was counted using point picker tool in ImageJ software. Data are presented as the percentage of fluorescence positive cells.

### Lipid peroxidation (LPO)

Lipid peroxidation assays were performed as described by Khan [19]. The results for the honokiol-treated groups were converted to the percentage of the control group.

### Measurement of protein carbonylation

Protein carbonylation was performed as described by Pushpanathan [20]. The results for the honokiol-treated groups were converted to the percentage of the control group.

### Measurement of antioxidant enzymes activities

The activities of antioxidant enzyme SOD, GPX, and CAT in *C*. *albicans* SC5314 were determined according to the manufacturer’s protocols [21]. The SOD, GPX, and CAT activities were all normalized by the protein content in each sample and converted to the percentage of the control group.

### Expression analysis of SOD genes in *C*. *albicans* cells

Following treatment of *C*. *albicans* SC5314 with 4, 8, 16 μg/mL of honokiol at 37°C for 12 h, the total RNAs were isolated using the hot phenol method, and then the real-time RT-PCR was performed as previously described [22]. Primer sequences of SOD1, SOD2, SOD3, SOD4, SOD5, SOD6 and ACT1 for amplification are shown in S1 Table.

### Analysis of mitochondrial membrane potential, morphology, and distribution

For determinations of mtΔψ, the fluorescent dyes JC-1 was used [23]. SC5314 cells were diluted to 1 × 10^7^ cells/mL in YPD medium with 16μg/mL honokiol at 37°C for 4h. Then cell suspensions were stained by 5 μmol/L JC-1 at 37°C for 30 min in the dark. The fluorescence of JC-1 (red fluorescence and green fluorescence) was monitored at Ex/Em = 490/525 nm and 490/590 nm with a fluorescence microscope (using FITC and TRITC filters) or a flow cytometer (using FL1 and FL2 channels).

To investigate the effect of honokiol on mitochondria morphology and distribution, 1 × 10^7^ cells/mL Tom70-GFP strain was treated by various concentrations of honokiol at 37°C for 4 h in YPD medium. The mitochondria morphology and distribution was visualized using confocal laser-scanning microscope (Olympus Fluoview FV1000) with 488nm excitation wavelength.

### Analysis of apoptotic markers

Protoplasts of *C*. *albicans* were stained with the Annexin V FITC apoptosis detection kit to assess cellular integrity and the externalization of phosphatidylserine as described earlier [24]. Representative picture of the images were taken using a fluorescence microscope.

### Statistical analysis

The data are presented as the means ± standard deviation (SD). Unpaired numerical data were compared using an unpaired t-test (two groups) or analysis of variance (ANOVA; more than two groups). A p value < 0.05 was considered significant.

## Results

### Honokiol induces intracellular ROS production

**Fig 1:**
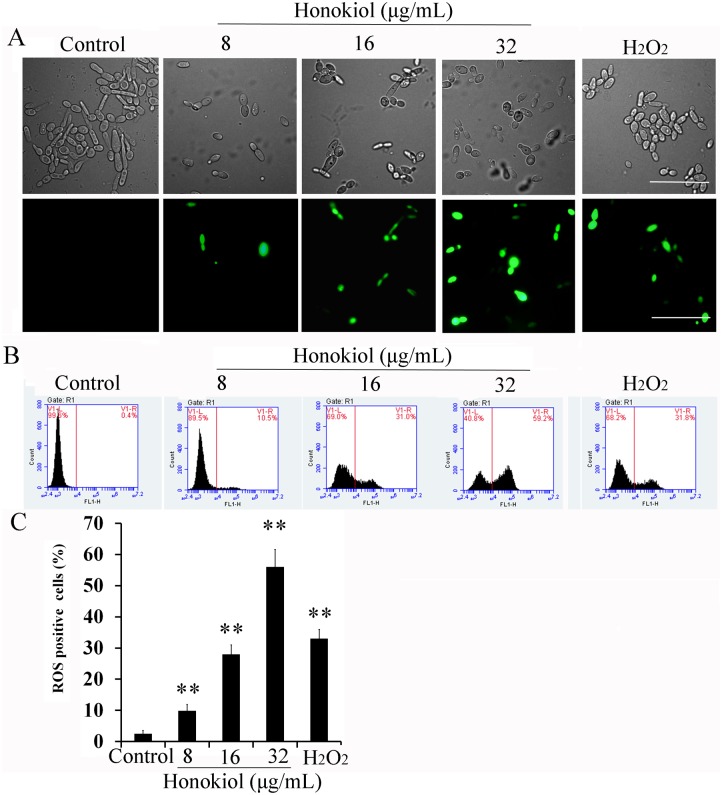
Honokiol induced ROS production in *C*. *albicans* SC5314. (A) ROS induction in *C*. *albicans* cells treated with honokiol observed by fluorescence microscopy. Scale bar = 20 μm. (B) Intracellular ROS in honokiol- treated *C*. *albicans* measured by flow cytometry. (C) The percentage of cells that produce ROS in the presence of different concentration of honokiol. 1.5 mmol/L of hydrogen peroxide (H_2_O_2_) was used as a positive control. The graph shows the average and standard deviation values. **, p<0.01.

**Fig 2:**
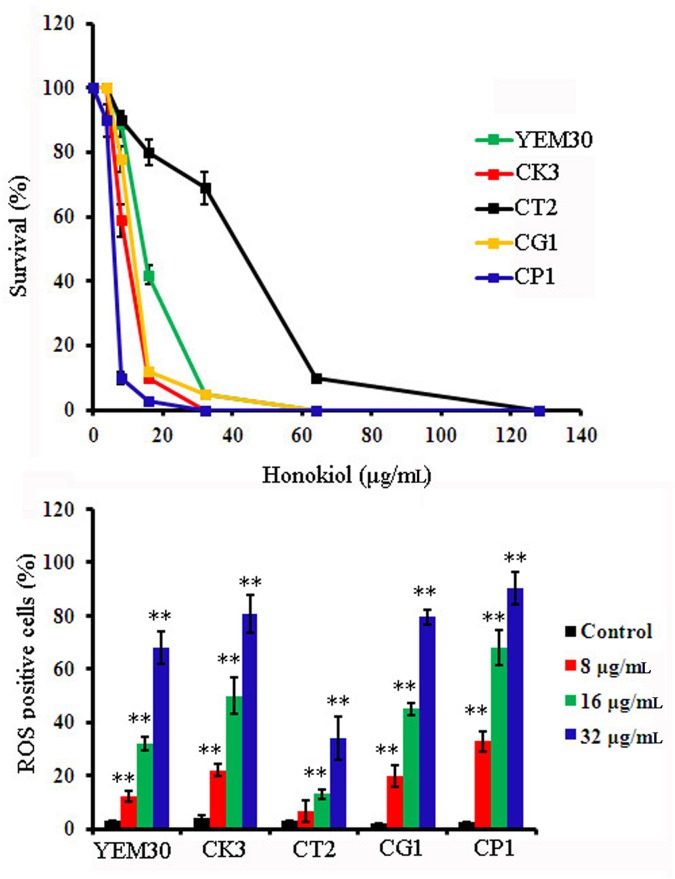
The percentage of survival cells (A) and ROS formation (B) after honokiol treatment in *Candida* species. (A) The percentage of survival cells was determined by measuring the optical density at 600 nm and then calculating the percentage of survival cells after compared to that of organisms grown in the absence of drug. (B) The percentage of cells that produce ROS of five *Candida* species after honokiol treatment was tested by flow cytometry. The graph shows the average and standard deviation values. **, p<0.01.

The fungicidal activity of antifungal compounds has classically been attributed to ROS production [5,7,9,11]. Both the minimum inhibitory and minimum fungicidal concentrations (MIC and MFC, respectively) for honokiol against *C*. *albicans* strain SC5314 were 32μg/mL [16]. Honokiol-induced generation of ROS in *C*. *albicans* SC5314 was quantified using DCFH-DA staining and indicated by DCF fluorescence due to DCFH-DA probe oxidation. As shown in Fig 1A, honokiol promoted cellular ROS generation in a dose-dependent manner. Quantification of cellular ROS generation in honokiol-treated *C albicans* cells by flow cytometry revealed a significant increase in DCF fluorescence after 4 h of incubation with honokiol. This effect was dose-dependent, as 9.9% of the cells showed ROS-positive staining after 8μg/mL of honokiol treatment, 28% showed ROS-positive staining after 16μg/mL of honokiol treatment, and more than 56% showed ROS-positive staining after incubation with 32μg/mL of honokiol (Fig 1B and 1C). At exogenous H_2_O_2_ concentrations of 1.5 mmol/L, fluorescent intensity was comparable to 16 μg/mL of honokiol treatment (Fig 1). A similar effect was also found when other *Candida* strains were treated with honokiol (Fig 2). Honokiol displayed marked antifungal activity against five tested *Candida* species. The honokiol MIC_80_ values against strains YEM30, CK3, CT2, CG1, and CP1 were 32, 16, 64, 16, and 8μg/mL, respectively. Notably, honokiol was more sensitive against strain CP1, where 8μg/mL of honokiol induced the most ROS when compared with other *Candida* strains. However, strain CT2 showed the least sensitivity to honokiol as it induced the least ROS, suggesting a clear relationship to its growth inhibitory effects (Fig 1A and 1B). Taken together, these results suggest that honokiol-induced eradication of *Candida* is likely due to ROS production.

### LPO and protein carbonylation

**Fig 3:**
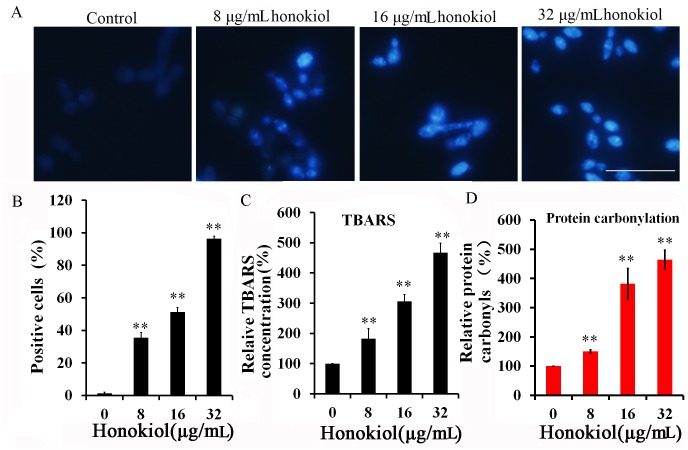
Honokiol-induced intracellular oxidation of lipids and proteins in *C*. *albicans* SC5314. (A) Fluorescence microscopic images taken after labeling of cells with DPPP. Scale bar = 50 μm. (B) The fluorescence positive cells was counted using point picker tool in ImageJ software. Data are presented as the percentage of fluorescence positive cells. (C) Concentration-dependent measurement of TBARS production in honokiol-treated *C*. *albicans* SC5314 by TBA assay. The results for the honokiol-treated groups were converted to the percentage of the control group. (D) Concentration-dependent measurement of protein carbonyls in honokiol-treated *C*. *albicans* SC5314 by DNPH assay. The results for the honokiol-treated groups were converted to the percentage of the control group. The graph shows the average and standard deviation of three different repetitions. **, p<0.01.

Irreversible oxidations of lipids and proteins have lethal effects on cells and lead to cell death. Therefore, ROS-derive oxidation of cellular proteins was investigated through analysis of *C*. *albicans* cell lysates treated with different honokiol concentration. The results clearly suggested a dose-dependent increase in the level of oxidation of lipids and proteins in honokiol- treated cells (Fig 3). DPPP is a compound that reacts with lipid hydroperoxides to produce the fluorescent product DPPP oxide. This reaction and its resulting fluorescent product were used to evaluate lipid peroxidation in viable cells. DPPP labeling of the cell membrane was verified using fluorescence microscopy (Fig 3A). Reaction of DPPP with hydroperoxides was determined by the increase in fluorescence intensity of the cell. The percent of DPPP positive cells was increased to 35.5%±3.2%, 51.2%±2.8%, and 96.3±1.5% when cells were treated with 8,16, and 32 μg/mL, respectively. Moreover, the oxidation of lipids by ROS was also investigated by measuring the absorbance of TBARS. TBARS levels were increased in honokiol-treated *C*. *albicans* cells in a dose-dependent manner as follows: 180.9%±33.1% for 8μg/mL of honokiol, 305.9%±23.3% for 16μg/mL, and 467.0%±32.2% for 32μg/mL, when compared with control (Fig 3B).

Finally, ROS-derived protein oxidation was studied in honokiol-treated *C*. *albicans* cells and a concentration-dependent increase in production of carbonyl groups was observed. The percent of protein carbonyls was increased to 150.3%±5.5%, 382.0%±52.4%, and 465%±31.5% when compared with DMSO control when cells were treated with 8, 16, and 32μg/mL of honokiol, respectively (Fig 3C). This observed elevation in both the level of protein carbonyls and TBARS in a concentration-dependent manner suggests that honokiol induced the oxidation of lipids and proteins.

### Antioxidant enzyme activities

**Fig 4:**
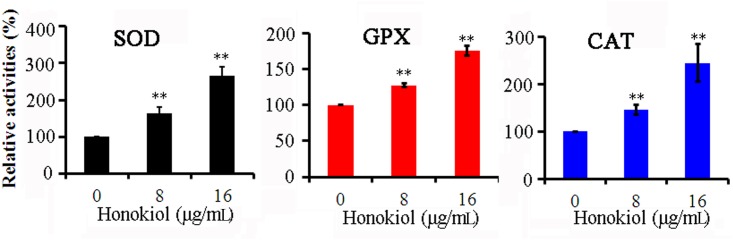
Effect of honokiol on the activities of antioxidant enzymes in *C*. *albicans* SC5314 cells. The activities of SOD, GPX, and CAT were determined as described above. Results show the average and standard deviation of three different repetitions. The results for the honokiol-treated groups were converted to the percentage of the control group. SOD, superoxide dismutase; GPX, glutathione peroxidase; CAT, catalase. **, p<0.01.

Next, we focused on the effects of honokiol on the activities of various antioxidant enzymes. Fig 4 shows the results of the relative activities of SOD, GPX, and CAT in *C*. *albicans* after treatment with different concentrations of honokiol. Honokiol treatment caused differential increases in antioxidant enzymatic activities. To this end, the activity of SOD significantly increased 1.6- and 2.6-fold in cells incubation with 8 and 16μg/mL honokiol, respectively (Fig 4A). Honokiol also increased GPX activity approximately 1.3- and 1.8-fold when treated with 8 and 16μg/mL honokiol, respectively (Fig 4B). As expected, CAT activity also increased 1.5- and 2.4-fold when cells were treated with 8 and 16μg/mL honokiol, respectively (Fig 4C).

### Honokiol upregulated SOD expression levels in *C*. *albicans* cells

**Fig 5:**
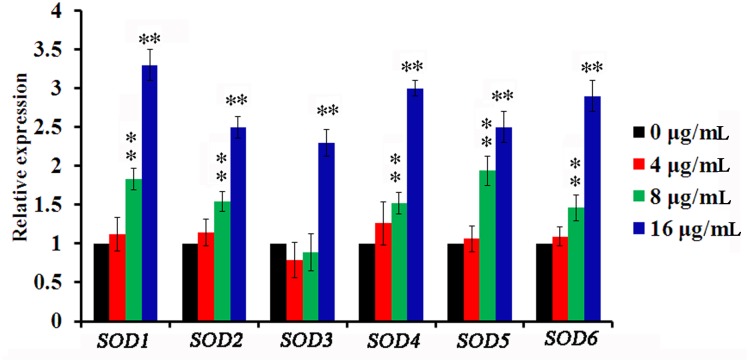
Relative SOD genes expression in honokiol-treated *C*. *albicans* compared to that control cells. SC5314 cells were treated with different concentration of honokiol, and the expressions of SOD genes were assessed using real-time PCR. Data represent the average and standard deviation for three independent experiments. **, p<0.01.

We next sought to estimate the effect of honokiol on the expression of SOD genes in *C*. *albicans* cells using real-time PCR. All SOD genes, except SOD3 (encoding manganese-containing superoxide dismutase), were significantly (P < 0.01) upregulated when treated with 8μg/mL honokiol when compared to the vehicle control (Fig 5). When treated with 16μg/mL honokiol, all SOD genes were upregulated between 2.3-fold (SOD3) and 3.3-fold (SOD1). Collectively, these data indicate the likely involvement of SOD in honokiol-induced oxidative stress responses.

### Potentiation of honokiol antifungal activity by SOD inhibition

**Fig 6:**
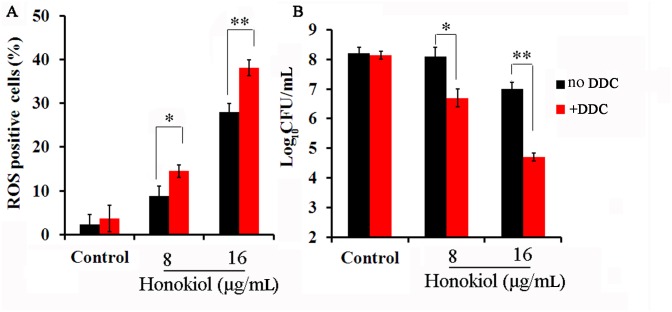
Effect of DDC on the ROS production (A) and Log_10_ CFU/mL (B) in *C*. *albicans* SC5314. (A) The percentage of cells that produce ROS of *C*. *albicans* after honokiol treatment was tested by flow cytometry. (B) Survival of *C*. *albicans* exposed to honokiol and DDC alone or in combination. Initial inoculum of SC5314 was 1.0 × 10^4^ cells/mL. After 24 h incubation, the suspension was serially diluted and plated on YPD agar for colony counts. Samples (100μl) were removed from the cultures and then diluted, plated, and incubated at 37°C for 48 h for colony counts. Results show the average and standard deviation of three different repetitions. *, P < 0.05; **, p<0.01.

We next sought to determine if treatment with the SOD inhibitor DDC enhanced ROS levels in honokiol-treated cells. To this end, SOD1, SOD4, SOD5, and SOD6 of *C*. *albicans* are Cu, Zn-containing SODs that can be inhibited by the copper chelator DDC [9]. It has been previously shown that when yeast cells are treated with DDC, SOD activities are inhibited in a concentration-dependent manner [9]. Moreover, 10 mmol/L DDC can inhibit 75% SOD activities *in vivo* [9]. However, in our study, DDC inhibited planktonic *C*. *albicans* growth *in vitro* at a concentration of 10 μmol/L (data not shown). Therefore, we assumed that the inhibitory effects of DDC on yeast growth were related to lower Cu, Zn-SOD activities. To ensure that DDC had a fungistatic not fungicidal effect, we chose 10 μmol/L DDC for this study. We next evaluated if pre-incubation with DDC increased the production of ROS after honokiol treatment. Pre-incubation for 1 h with DDC followed by honokiol at tested concentrations resulted in increased ROS levels in *C*. *albians* cells when compared to honokiol treatment alone (Fig 6A). Moreover, a combination of honokiol and DDC had a strong inhibitory effect that diminished the number of live cells by over 25- and 200-fold for 8 and 16μg/mL honokiol, respectively (Fig 6B).

### Honokiol alters mitochondrial membrane potential, morphology, and distribution

**Fig 7:**
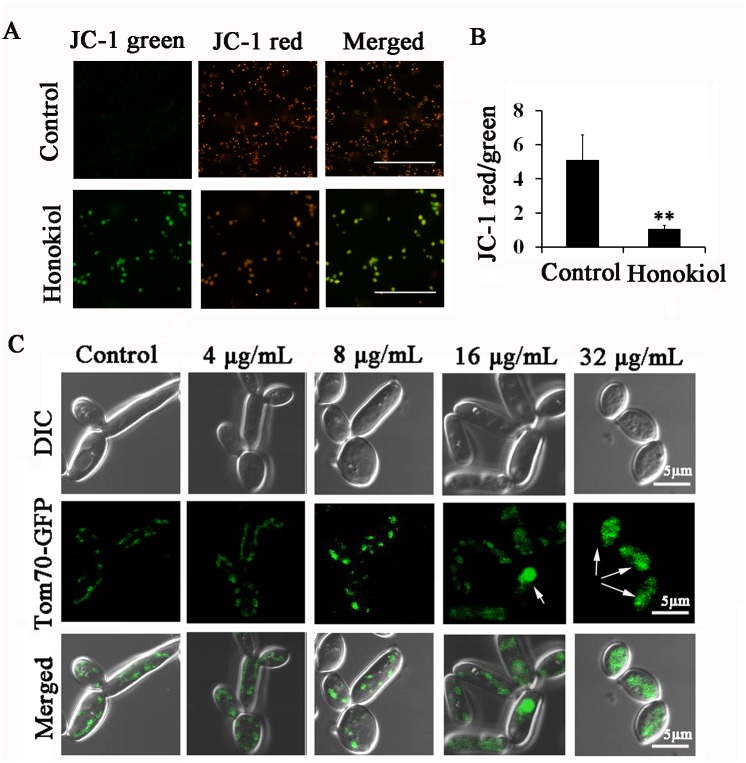
Honokiol treatment alters mitochondrial membrane potential, morphology, and distribution. (A) Measurement of mtΔψ in honokiol-treated *C*. *albicans* cells observed by fluorescence microscopy. Scale bar = 20 μm. (B) The effect of honokiol on the ratio of JC-1 green/red was measured by flow cytometry. Results show the average and standard deviation of three different repetitions. **, p<0.01. (C) Mitochondrial morphology and distribution were altered by honokiol treatment. Scale bar = 5 μm.

MtΔψ is essential for normal mitochondrial functions, including protein synthesis and import in addition to ATP production [6]. In this study, the effect of honokiol on mtΔψ was measured by fluorescence microscopy and flow cytometry using JC-1 staining. As shown in Fig 7A, the control group displayed a typical fluorescence distribution of JC-1, with the formation of J-aggregates (red) in mitochondria. Interestingly, after 4 h incubation with 16μg/mL honokiol, cells stained with JC-1 had dramatically changed fluorescence patterns as indicated by the cytoplasmic formation of J-monomeric (green) forms. Moreover, incubation with honokiol also resulted in a significantly reduced ratio of red to green fluorescence of JC-1 (Fig 7B).

Relatedly, we were also interested in determining whether honokiol treatment affected mitochondrial morphology and organization in *C*. *albicans*. We used a fusion of the outer mitochondrial membrane protein Tom70 with green fluorescent protein (Tom70-GFP) to visualize mitochondrial morphology and distribution. Consistent with previous description of normal yeast mitochondria, control cells appeared either as multiple small dots concentrated around the edge or as a tubular structure extending along the peripheral cytoplasm(Fig 7C). In contrast, honokiol treatment altered mitochondrial morphology and distribution, as shown by Tom70-GFP localization. To this end, both 16μg/mL or 32μg/mL honokiol treatment resulted in clusters formation in the central cytoplasm (Fig 7C). These results indicated that honokiol treatment leads to alter mitochondrial morphology, localization, and functioning.

### Honokiol induces apoptosis

**Fig 8:**
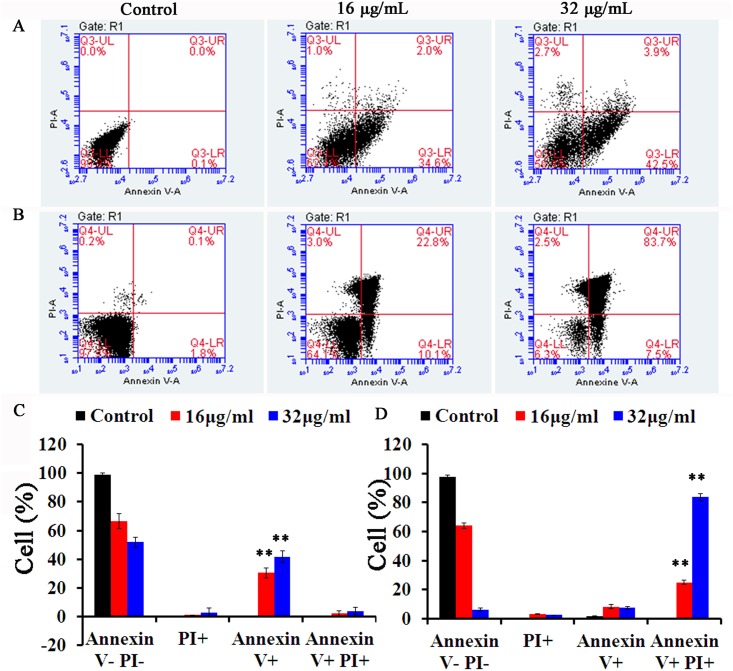
Externalization of phosphatidylserine in the presence of honokiol in *C*. *albicans* SC5314. After treatment for 4 h (A) or 24 h (B), the cells were analysed by flow cytometry as described in Materials and methods. In each plot, the indication for watch quadrant is as follow: lower left: viable cells (annexin- PI-); lower right: early apoptotic cells (annexin+ PI-); upper right: late apoptotic cells (annexin+ PI+); upper left: necrotic cells (annexin- PI+). (B) The percentage of apoptotic cells after treatment for 4 h (C) or 24 h (D) was determined by flow cytometry. Results show the average and standard deviation of three different repetitions. **, p<0.01.

Increased ROS levels have always been considered to play a key role in mediating programmed cell death (PCD) like apoptosis [24]. Given this, we examined the extent of phosphatidylserine externalization in *C*. *albicans* by using flow cytometry in conjunction with FITC-labeled annexin V and the membrane impermeant fluorochrome PI. In this assay, apoptotic cells were able to bind to annexin V-FITC after digestion of the cell wall, whereas necrotic cells accumulated only PI (PI+). The percentage of annexin V (+) PI (-) (early apoptotic cells) at the basal level was 0.1% in cells treated with the control vehicle only. When cells were treated with honokiol at either 16μg/mL or 32μg/mL for 4 h, the percentage of early apoptotic cells increased to 30.5% and 41.6%, respectively (Fig 8A and 8C). In addition, we also examined the effect of 24 h treatment with honokiol on SC5314 cellular apoptosis. The total proportion of apoptotic cells, including those in early and late stages, was increased in a concentration-dependent manner. After 24 h incubation with 32μg/mL honokiol, the majority of cells were at late stage of apoptosis (83.7%, Fig 8B and 8D).

## Discussion

*Candida* species are a group of opportunistic fungal pathogens in humans, particularly among immunocompromised population [2,25,26]. Due to the limited numbers of clinical antifungal agents, serious side effects, toxicity, as well as the development of resistant strains, the treatment of candidiasis faces severe clinical challenges [3]. However, several molecules originating from natural plants have shown obvious antifungal activities and are a potential source for the discovery of novel anti-*Candida* therapies [10,27,28]. To this end, previous works examining the anti-Candida activities of phenolic compounds have shown their ability for enzymatic inactivation as well as inhibition of biofilm formation [10]. Past studies have also demonstrated that honokiol has antifungal activities (MIC rang, 16–32 μg/mL) and when used in combination with fluconazole, can exhibit synergistic activity against clinical isolates of fluconazole-resistant *C*. *albicans* [16,29,30]. Due to concerns about poor aqueous solubility, liposomal formulations of honokiol have been developed [31]. These formulations maintained honokiol plasma concentration of above 30μg/mL for 24 h after intravenous injection of 25 mg/kg liposomal honokiol [31]. This result demonstrated that effective inhibitory concentrations are achievable *in vivo* [31]. Honokiol toxicity was also assessed in rats by intravenous administration of doses ranging from 20–80mg/kg body weight. There were no significant differences between the honokiol-treated group and vehicle-treated groups, lending support to the safety of in vivo use of honokiol [32]. To better understand how honokiol works on a cellular level, we sought to explore its antifungal mechanism.

The chemical structure of honokiol contains two phenolic groups, which may exhibit antioxidant properties like vitamin C and E [33]. It has been reported that honokiol could act as a strong ROS scavenger in either a cell-free system or in melanoma cells [34]. Interestingly, in this study, honokiol caused a significant increase in ROS accumulation in all tested *Candida* species (*C*. *albicans*, *C*. *tropicalis*, *C*. *glabrata*, *C*. *parapsilosis*, and *C*. *krusei*) (Figs 1 and 2). This result suggests that ROS generation acts as an important factor in determining honokiol cytotoxicity. Some researchers have hypothesized paradoxical pro- and antioxidant activities with honokiol treatment [35]. However, the exact mechanisms for pro- and antioxidant activities of honokiol remain unclear. Similar results were also obtained from vitamin C and E, which have also been reported to have both antioxidant and pro-oxidant activities [36].

The elevation of intracellular ROS ultimately induces oxidative damage to proteins, lipids, and DNA. Failure to suppress such damage is linked with cell death [6]. The plasma membrane of *C*. *albicans* contains approximately 70% polyunsaturated lipids. Consequently, the formation of lipid peroxides will lead to structural and functional deformities of the membrane [37]. ROS reactions with proteins can cause the formation of protein cross-links as well as oxidation of both the peptide backbone and amino acid side chains [6]. To this end, the TBARS assay is the most popular biochemical assay to quantify lipid hydroperoxides using absorption spectroscopy [38]. In addition, fluorescence staining provides an additional, more sensitive, measure [39]. DPPP is highly lipophilic and selectively reacts with lipid hydroperoxides. Importantly, it does not react with aqueous peroxides, which is in contrast to the fluorescent probe DCFH-DA [39]. It has been shown that cellular DPPP shows remarkably higher reactivity toward MeLOOH when compared with H_2_O_2_ [39]. The results of our DPPP staining and TBARS assay indicated an increase in the level of LPO induced by honokiol treatment, suggesting significant generation of ROS (Fig 3). Consistently, honokiol-induced protein carbonylation was increased in a dose-dependent manner, which may be associated with the increase of oxidative stress inside the cells (Fig 3D).

Yeast cells contain limited pools of antioxidants that can sufficiently protect against ROS. However, these default states of defense cannot safeguard the cells from sudden oxidative insults. The potent induction of SOD, GPX, and CAT activities in reaction to elevated levels of ROS play a key role in the antioxidant defense system for aerobic organisms [40]. Prominent among these are Mn-SOD and Cu, Zn-SOD, which scavenge O_2_^•−^ and transform it to H_2_O_2_ [40]. GPX is susceptible to any increase in H_2_O_2_, while CAT responds with further degradation of H_2_O_2_ to water [40]. Activities of the tested primary defense enzymes increased significantly in honokiol-treated cells (Fig 4). ROS generation stimulated antioxidant pools including those containing SOD, GPX, and CAT to counteract its damaging effects. In response to honokiol-induced oxidative stress, several SOD genes were upregulated (Fig 5). SOD1, SOD4, SOD5, and SOD6 of *C*. *albicans* were inhibited using the Cu, Zn-SOD inhibitor DDC [9]. Based on our data, the SOD inhibitor DDC was able to reduce the antioxidative capacities of *C*. *albicans* cells, resulting in increased efficacy of honokiol against *C*. *albicans* (Fig 6). However, the rapid upregulation and high efficiency of yeast antioxidant defenses still cannot protect cell biomolecules from severe oxidative damage caused by honokiol.

Mitochondria are indispensable for cellular energy production by the tricarboxylic acid cycle and conserved processes like iron metabolism [6]. In this study, honokiol treatment altered mitochondrial morphology, distribution, and membrane potential (Fig 7). To reach this conclusion, we used JC-1 for cytofluorimetric analysis of mtΔψ. The ratio of red to green fluorescence of JC-1 is dependent only on membrane potential, and not influenced by mitochondrial size, shape, or density [23]. After honokiol treatment, the ratio of red to green JC-1 fluorescence of was significantly reduced, indicating mitochondrial depolarization. We also used rhodamine 123 (Rh123) for mtΔψ estimation. In contrast to JC-1, Rh123 accumulation was observed after honokiol treatment, indicating an increase in mtΔψ (data not shown). Rh123 fluorescence was altered independently of mtΔψ, elicited either by the activity of multidrug resistance pumps for Rh123 or changes to the plasma membrane potential [41]. Honokiol or magnolol could compete with the ABC transporter Cdr1p substrates, thereby increasing Rh123 intracellular accumulation [22]. In view of its interference with this efflux pump, Rh123 staining was not suitable for mtΔψ estimation in this study. By monitoring a fluorescently tagged outer mitochondrial membrane protein Tom70-GFP, we found that mitochondrial morphology and distribution were altered after honokiol treatment. These findings suggest that honokiol may induce mitochondria dysfunction in *C*. *albicans*.

Mitochondria have always been considered as a primary source of ROS like the superoxide anion radicals, which are converted from O_2_ by electron leakage from the mitochondrial transport chain during cellular respiration [6]. However, we are still lacking a detailed mechanistic knowledge of the architecture of mitochondrial ROS-generation systems induced by honokiol such as that of respiratory chain complex I or complex III. Thus, future work will aim to clarify specific mitochondrial ROS-generation components that are activated after honokiol treatemt. The excess ROS generated damages mitochondrial functioning and causes the necrosis or apoptosis of *C*. *albicans*. To this end, our work has shown that 4h or 24 h of honokiol treatment results in higher levels of apoptosis in *C*. *albicans* (Fig 8).

## Conclusion

In summary, our study suggests that ROS accumulation may be an important factor responsible for the fungicidal activity of honokiol. Our results also show that ROS production by honokiol is associated with mitochondrial dysfunction.

## Supporting information

S1 Table Gene-specific primers used for real-time RT-PCR. (DOC)
