# Supplementary material for: Honokiol induces reactive oxygen species-mediated apoptosis in Candida albicans through mitochondrial dysfunction
Source: PLoS One. 2017 Feb 13;12(2):e0172228. doi: 10.1371/journal.pone.0172228 (PMC5305218; doi:10.1371/journal.pone.0172228)
Supplement: S1 Table — (DOC) [file pone.0172228.s001.doc]

**Supplementary Information**

Supporting Information legends:

S1Table. Gene-specific primers used for real-time RT-PCR.

S1 Table Gene-specific primers used for real-time RT-PCR.

| Genes | Strain | Genotype |
| --- | --- | --- |
| *ACT1* | F | TTTCATCTTCTGTATCAGAGGAACTTATTT |
|  | R | ATGGGATGAATCATCAAACAAGAG |
| *SOD1* | F | TCCGAATCCGCTCCAACCACA |
|  | R | AAATGAGGACCAGCAGAAGTACAACCA |
| *SOD2* | F | TCAATTGAACAAGCCGTTGAAGCCAAA |
|  | R | ACCACCTTGAGAGACAGGAGCCA |
| *SOD3* | F | CAATGCCGCTATTGACGCACTTGA |
|  | R | TCCAGAACAAACTGTGGTTGGTGTGT |
| *SOD4* | F | TGACTCCAAAGGCAAGGCACCA |
|  | R | TGGGCCAACACCTGAAGGCAAT |
| *SOD5* | F | ACGAGGGACACGGCAATGCT |
|  | R | GCGCCATTACCTTGAGGAGCAGTA |
| *SOD6* | F | GACCCCGACCCACCTCAACAA |
|  | R | GGGTAGCAAGGAGTGCCGGT |
